# The Efficacy of Clinical Breast Exams and Breast Self-Exams in Detecting Malignancy or Positive Ultrasound Findings

**DOI:** 10.7759/cureus.22464

**Published:** 2022-02-21

**Authors:** Nicole Huang, Lu Chen, Jing He, Quan D Nguyen

**Affiliations:** 1 Internal Medicine, University of Texas (UT) Dell Medical School, Austin, USA; 2 Biostatistics and Epidemiology, University of Texas Medical Branch (UTMB), Galveston, USA; 3 Pathology, University of Texas Medical Branch (UTMB), Galveston, USA; 4 Radiology, Baylor College of Medicine, Houston, USA

**Keywords:** breast-radiology-mammography-ultrasound-mri-biopsyelastography, clinical breast exams, breast self-exams, breast cancer, breast lumps

## Abstract

Objective

A breast exam is a low-risk, low-cost method for early detection, which is crucial for improved mortality. However, clinical breast exams (CBE) and breast self-exams (BSEs) remain controversial with unclear guidelines. This study analyzes the efficacy of these two exam types in evaluating palpable breast masses.

Methods

This retrospective cross-sectional study included 2019 medical records from Epic of women with breast lumps. Patient demographics, provider types, and breast exam types were recorded. Primary outcomes were detection of cancer and positive ultrasound finding. Fisher’s exact tests and two-sample t-tests determined the statistical significance of the association between the outcomes and categorical and continuous variables.

Results

Of 462 breast masses, 69 demonstrated positive ultrasound findings, with 26 of those yielding cancer; 96% of cancers and 81% of ultrasound findings resulted from patient-identified lumps. Of provider-identified lumps, 100% of cancers and 92.3% of positive ultrasound findings were diagnosed by MDs (doctors of medicine) rather than midlevel providers. There was no statistically significant difference in identifying cancer or positive ultrasound finding between CBEs and BSEs (p = 0.3709 and p = 0.1556).

Conclusion

Despite no difference between CBEs and BSEs in identifying cancer or positive ultrasound finding, 25 of the 26 breast cancers were initially detected by patients, while only one of 26 was detected by CBE. BSEs detect breast cancers. Although some guidelines encourage CBEs over self-exams, not all CBEs are equal.

Key message

There is no significant difference between CBEs and BSEs in identifying cancer or positive ultrasound finding. The majority of cancers were initially identified by patients. BSEs detect breast cancers and women should continue performing them. Not all CBEs are equal. CBEs by MDs, especially women health specialists, are generally more effective than those by midlevel providers.

## Introduction

Breast cancer is the leading cancer in American women, aside from skin cancer. A woman is diagnosed with breast cancer in the United States every two minutes. Over a lifetime, one in eight women in the United States will develop breast cancer. If the cancer is diagnosed early and remains localized, treatment is more effective, and the five-year survival rate is 99% [1]. Thus, early detection is crucial for improved mortality. Tools for early detection include clinical breast exams, breast self-exams, and mammograms. Compared to imaging, breast exams are of lower risk and more cost-effective. A clinical breast exam (CBE) is performed by a medical provider, while a breast self-exam (BSE) is performed by the patient. In less-developed countries where imaging and CBEs are not as readily available, BSEs are often the only method for early detection of breast cancer [2]. Even in the United States, BSEs are valuable for women who are younger than the age of 40 or 50 years, the age at which mammography is recommended (depending on the guideline), and who are at higher risk of mortality from breast cancer due to race or family history [3].

Current guidelines regarding CBEs and BSEs, however, are controversial. The American Cancer Society no longer recommends CBEs or BSEs for women with an average risk for breast cancer because research has not associated them with clear benefits in settings where mammography screening is available and awareness is high. However, they suggest women to remain familiar with their breasts and report changes to health providers [4]. The U.S. Preventive Services Task Force also discourages BSEs and states that there is not enough evidence to evaluate CBEs [5]. Only a few organizations still fully endorse the use of CBEs. The National Comprehensive Cancer Network recommends CBEs every one to three years from age 25 to 39 years and annually after the age of 40 years [6].

Despite guidelines, literature seems to suggest that CBEs are better than BSEs [7-10]. While one Swiss study showed no significant difference in the detection of tumors between CBEs and BSEs, the authors still recommended CBEs for older women [11]. Some articles even suggest that CBEs be included in addition to mammography to increase the sensitivity of breast cancer screening [12]. In one meta-analysis, CBEs detected cancer with a sensitivity and specificity of 54% and 94%, respectively [13].

The purpose of this paper is to examine the efficacy of CBEs and BSEs in evaluating palpable breast masses and identifying malignancies or any positive ultrasound results. We are interested in ultrasound results because ultrasound is used to assess breast lumps in women of all ages. Mammograms, due to radiation exposure, are reserved for use in women older than the age of 30. Lehman et al. proposed the protocol for imaging abnormal breast lumps to use ultrasound as “the primary imaging tool for women who are pregnant, lactating, or younger than 30 years. For women who are 40 years old and older, mammography, followed in most cases by ultrasound, is recommended. For women of 30-39 years old, ultrasound or mammography may be performed first at the discretion of the radiologist or referring provider” [14].

Our research is novel as no previous publication in the United States has compared the efficacy of CBEs to BSEs. We hope that our results can contribute to modern breast cancer screening guidelines. We hypothesize that BSEs will be better than CBEs in evaluating breast lumps as women are more familiar with the changes in their own breasts than physicians. Furthermore, not all providers are trained to adequately perform CBEs.

## Materials and methods

This retrospective chart review of 462 women of all ages with palpable breast lumps was collected from Epic between January 1, 2019, and December 31, 2019. The keywords such as “breast mass,” “breast lumps,” and “breast nodules” were used in the computer search. Lumps identified by patients were categorized as BSEs, whereas those found by physicians were classified as CBEs, regardless if a CBE was also performed after a BSE to further evaluate the patients’ findings. Most CBEs, however, were performed for asymptomatic patients with a history of breast cancer. This study was approved by the Institutional Review Board of the University of Texas Medical Branch (IRB#: 19-0277) who granted a waiver of consent. This study was compliant with the Health Insurance Portability and Accountability Act. Patient age, personal history of breast cancer, family history of breast or ovarian cancer, and breast exam type (CBE or BSE) were recorded. For those who had lumps identified by providers, referring provider type (doctor of medicine [MD], doctor of osteopathic medicine [DO], or others) and referring clinical unit (Galveston, League City, or Angleton Danbury Campus [ADC]) were also recorded. Primary outcomes were detection of cancer and positive ultrasound finding, both defined using the American College of Radiology’s Breast Imaging Reporting and Database System (BI-RADS) Atlas (Categories 0-6). Positive ultrasound findings included Categories 2-5 [15].

The statistical significance of association between the outcomes and categorical variables was tested using Fisher’s exact tests, and continuous variables were tested using two-sample t-tests. All statistical tests were two-sided, and p < 0.05 was considered significant. All analyses were performed using SAS statistical software, version 9.4 (SAS Institute, Cary, North Carolina).

## Results

The majority of the breast lumps (84.6% of 462) in the study were identified by patients (Tables 1, 2). Further evaluation of these masses revealed 69 positive ultrasound findings with 26 of those yielding cancer, and 10 of them were classified as early-stage (stage 0 and 1). Of the cancers and ultrasound results, most (96% and 81.2%) were reported by patients. Regarding lumps that were identified by providers, 100% of the diagnosed cancers and 92.3% of the positive ultrasound findings were discovered by MDs rather than DOs or midlevel practitioners. Only one of 13 positive ultrasound findings was detected by midlevel providers (Table 2). Furthermore, 100% of the diagnosed cancers were associated with the League City clinic, whereas 76.9% of the positive ultrasound findings were diagnosed in the League City clinic and 23.1% in the Galveston clinic. The Galveston clinic includes the Correctional Managed Care (CMC) facility where the referring providers are generally midlevel practitioners (nurse practitioners and physician assistants). However, there was no statistically significant difference between the clinic locations and the outcomes of cancer or positive ultrasound findings (p = 1 or p = 0.6416). Although most of the palpable masses were identified by patients themselves, there was no statistically significant difference in BSEs compared to CBEs in identifying cancer or positive ultrasound findings (p = 0.1556 and p = 0.3709).

**Table 1:** Association of each variable with detection of cancer BSE: Breast self-exam; CBE: clinical breast exam; MD: doctor of medicine; DO: doctor of osteopathic medicine; ADC: Angleton Danbury Campus.

Variables	Total (N = 462)	No Cancer (N = 435)	Cancer (N = 26)	P-values
Age (Mean, SD)	42.02 (15.52)	40.98 (14.91)	57.77 (14.92)	
Breast exam type				0.1556
BSE	391 (84.63)	365 (83.91)	25 (96.15)	
CBE	71 (15.37)	70 (16.09)	1 (3.85)	
Referring provider type				1
MD	50 (71.43)	49 (71.01)	1 (100.00)	
DO	3 (4.29)	3 (4.35)	0	
Others	17 (24.29)	17 (24.64)	0	
Referring clinical unit				
Galveston	23 (32.39)	23 (32.86)	0	1
League City clinic	45 (63.38)	44 (62.86)	1 (100.00)	
ADC	3 (4.23)	3 (4.29)	0	
Family history				0.3323
Breast	102 (22.32)	99 (22.92)	3 (12.00)	
Breast and ovarian	8 (1.75)	7 (1.62)	1 (4.00)	
Ovarian	5 (1.09)	5 (1.16)	0	
None	342 (74.84)	321 (74.31)	21 (84.00)	
Personal history of breast cancer				0.5749
No	443 (96.72)	419 (96.77)	24 (96.00)	
Yes	15 (3.28)	14 (3.23)	1 (4.00)	

**Table 2:** Association of each variable with detection of positive ultrasound finding BSE: Breast self-exam; CBE: clinical breast exam; MD: doctor of medicine; DO: doctor of osteopathic medicine; ADC: Angleton Danbury Campus.

Variables	Total (N = 462)	No finding (N = 392)	Positive ultrasound finding (N = 69)	P-values
Age (Mean, SD)	42.02 (15.52)	41.08 (14.57)	46.72 (18.83)	0.0202
Breast exam type				0.3709
BSE	391 (84.63)	334 (85.20)	56 (81.16)	
CBE	71 (15.37)	58 (14.80)	13 (18.84)	
Referring provider type				0.2735
MD	50 (71.43)	38 (66.67)	12 (92.31)	
DO	3 (4.29)	3 (5.26)	0	
Others	17 (24.29)	16 (28.07)	1 (7.69)	
Referring clinical unit				0.6416
Galveston	23 (32.39)	20 (34.48)	3 (23.08)	
League City clinic	45 (63.38)	35 (60.34)	10 (76.92)	
ADC	3 (4.23)	3 (5.17)	0	
Family history				0.4704
Breast	102 (22.32)	91 (23.39)	11 (16.18)	
Breast and ovarian	8 (1.75)	7 (1.80)	1 (1.47)	
Ovarian	5 (1.09)	5 (1.29)	0	
None	342 (74.84)	286 (73.52)	56 (82.35)	
Personal history of breast cancer				0.4693
No	443 (96.72)	379 (96.93)	64 (95.52)	
Yes	15 (3.28)	12 (3.07)	3 (4.48)	

Most patient characteristics, such as personal history of breast cancer or family history of breast or ovarian cancer, had no significant effect on the outcome of lumps being cancer or positive ultrasound findings. The majority of 462 patients had no personal history of breast cancer (96.7%) or family history of breast or ovarian cancer (74.8%). Of the 26 patients with cancer, 96% had no personal history of breast cancer and 84% had no family history of breast or ovarian cancer (p = 0.5749 and p = 0.3323). Table 2 shows that out of 69 positive ultrasound findings, 95.5% had no personal history of breast cancer and 82.4% had no family history of breast or ovarian cancer (p = 0.4693 and p = 0.4704). Age, on the other hand, was statistically significant among the patients with palpable breast masses that were associated with cancer or positive ultrasound findings (p < 0.0001 and p = 0.0202). The average age of 462 patients was 42. Patients with cancer or positive ultrasound findings were older on average (57.8-years old with an SD of 14.9 and 46.7-years old with an SD of 18.8, respectively).

Interestingly, of the 26 patients with cancer, only six had received prior screening mammograms. Annual screening mammograms allow for early detection of breast cancers before they become palpable. It is possible that if the other 20 patients had annual screening mammograms, some of those cancers may have been detected earlier before becoming palpable.

## Discussion

This study’s main result was that the efficacy of detecting malignancy or any positive ultrasound finding did not differ significantly between CBEs and BSEs. Both exams were of similar values. However, most cancers and positive ultrasound findings were identified by patients. If cancers were diagnosed by providers, the providers were usually physicians rather than midlevel practitioners (nurse practitioners and physician assistants).

Our finding of the importance of BSEs is consistent with prior studies, which also showed breast cancers to be patient-identified. Roth et al. demonstrated that 39% of breast cancers in their study were detected by patients. In breast cancer survivors, the rate was 25%. Only 43% of the study’s breast cancers were diagnosed with mammography [3]. In another study, two-thirds of women self-detected their breast cancer [16]. Szukis et al. reported that having a history of performing BSEs was associated with increased detection rates of breast cancer [17]. This suggests that the habit of conducting BSEs is beneficial. Some guidelines discourage BSEs due to false-positive lumps causing more emotional harm than good [18]. Numerous imaging tests, biopsies, and surgeries lead not only to anxiety but also to financial consequences. The cost of positive BSEs is further workup with imaging studies including diagnostic mammograms and breast ultrasounds. According to the Susan G. Komen Foundation, the cost in Texas for diagnostic imaging with private insurance ranges from $336 to $836, and for uninsured women, the cost could be more than $1,000 out-of-pocket. The benefit of detecting cancer on BSEs is the lower cost of treatment when diagnosing at an earlier stage. Comparing five-year survival rates, stages zero and one are 100%, stage two is 93%, stage three is 72%, and stage four is 22% [19]. Recommendations against BSEs are mainly based on the results of an outdated Shanghai trial that took place more than 30 years ago. The study concluded that BSEs were not associated with reductions in breast cancer mortality [20]. Since then, literature on this topic has been scarce. Based on this article, BSE is a skill that women should continue to practice because it diagnoses breast cancer. With early detection correlating with reduced mortality, it is possible that BSEs could have a significant impact on breast cancer mortality.

The data from this paper also insinuates that CBEs when performed by physicians specializing in women’s health, such as breast surgeons or obstetrics and gynecology (OB-GYN) doctors, are more effective than those performed by general practitioners. This could be explained by many physicians’ lack of training in CBE skills and discomfort in performing these exams [21]. One study showed that physicians who were male, of family medicine specialty, and perceptive of patient embarrassment had lower rates of CBE [22]. Perhaps for CBEs to be more effective tools in detecting cancer, physicians need to first overcome their unease and then hone their CBE skills. More standardization in CBE techniques among providers could increase its efficacy in detecting malignancies.

Limitations

Limitations of this study include its retrospective design that involved chart review. Some charts, mostly of patients in the prison hospital (CMC location), were missing information about the personal history of breast cancer, family history of breast or ovarian cancer, and whether or not family members with cancer were first- or second-degree. Perhaps those with more first-degree compared to second-degree relatives would have been more inclined to seek a health provider for evaluation of their breast mass. On the other hand, perhaps, patients with a history of benign breast lumps would have been less concerned about subsequent breast masses and not sought further evaluation. Another piece of information that could not be extracted from charts is the final specialty for interns. This could have affected the attribution of providers to certain departments. For example, if a surgical intern was rotating through internal medicine when he/she identified a patient’s breast mass, then the identifying provider would have been mislabeled as internal medicine rather than surgery. While the study lost eight of 421 patients to follow-up (no post-imaging biopsy results), these patients were not excluded. Their breast masses were classified as not cancer as all of these masses received BI-RADS 4A on imaging, an indication of less than 10% chance of malignancy [23].

Other limitations of the study include differing definitions of BSE and CBE as there is no consensus on one specific method for breast exams. So, patients and physicians may employ different techniques for evaluating palpable masses. Lastly, this study may not be generalized to all women in America as patient charts were taken from tertiary care centers in Galveston, Texas. The racial, ethnic, and socioeconomic distributions of these patients may differ from other populations.

## Conclusions

In summary, in patients presenting for evaluation of breast lumps, there is no significant difference between CBEs and BSEs. However, the majority of breast cancers are detected by patients. This suggests that BSEs are important and do detect cancer. Despite controversial guidelines, women should continue to perform BSEs for early breast cancer screening. Although some guidelines encourage CBEs over BSEs, not all CBEs are equal. CBEs by MDs, especially women health specialists like breast surgeons and OB-GYN physicians, are generally more effective than those by midlevel providers.
